# NKG2D signaling certifies effector CD8 T cells for memory formation

**DOI:** 10.1186/s40425-019-0531-2

**Published:** 2019-02-18

**Authors:** Cynthia Perez, Kushal Prajapati, Brianna Burke, Lourdes Plaza-Rojas, Nancy J. Zeleznik-Le, Jose A. Guevara-Patino

**Affiliations:** Oncology Institute, Cardinal Bernardin Cancer Center, Loyola University Chicago, Maywood, IL USA

**Keywords:** Immunological memory, NKG2D, CD8 T cells, Mouse models, Memory formation

## Abstract

**Background:**

The development of memory responses is an evolutionary function of the adaptive immune system. We propose that for the immune system to populate the memory compartment with the best-suited CD8 T cells it utilizes a process of certification or molecular accreditation mediated through Natural Killer Group 2D (NKG2D). This process of certification assures that the memory compartment is filled with CD8 T cells that have demonstrated their ability to kill their cognate targets through a two-step process that utilizes T cell receptor (TCR) and NKG2D signaling.

**Methods:**

One week after immunization with peptide-pulsed dendritic cells, NKG2D signaling was transiently blocked in vivo with a single injection of neutralizing antibodies. Under such conditions, we determined the importance of NKG2D signaling during the effector phase for memory formation without compromising NKG2D signaling at the memory phase**.** Both open (polyclonal) and closed (monoclonal) CD8 T cell repertoires were studied.

**Results:**

We show that signaling through NKG2D mediated this certification. Temporary blockade of NKG2D signaling during the effector phase resulted in the formation of highly defective memory CD8 T cells characterized by altered expression of the ribosomal protein S6 and epigenetic modifiers, suggesting modifications in the T cell translational machinery and epigenetic programming. Finally, these uncertified memory cells were not protective against a B16 tumor challenge.

**Conclusion:**

Signaling through NKG2D during the effector phase (certification) favors the development of functional memory CD8 T cells, a previously undescribed role for NKG2D. Temporary blockade of NKG2D signaling during the effector phase results in the formation of highly defective memory CD8 T cells potentially by affecting the expression of the ribosomal protein S6 and epigenetic modifiers, suggesting alterations in T cell translational machinery and epigenetic programming.

**Electronic supplementary material:**

The online version of this article (10.1186/s40425-019-0531-2) contains supplementary material, which is available to authorized users.

## Introduction

Vaccination and T cell-based immunotherapy rely on one important feature of the adaptive immune system: the ability to form long-lasting memory cells able to rapidly respond to a second exposure with the same antigen [1–3]. While the different phases of an immune response are well known, the mechanisms underlying the formation of protective memory are not well understood.

Several factors can influence memory formation. Interleukin-15 (IL-15) is one the most well described factors needed for survival and homeostasis of memory and memory precursor cells, as blocking IL-15 signaling strongly reduces the number of memory CD8 T cells [4, 5]. Transcription factors, such as T-box transcription factor (T-bet) and T cell factor-1 (Tcf-1), also control memory formation [6–8]. Repressing T-bet expression favors memory differentiation [8], while Tcf-1 is upregulated in memory CD8 T cells [6, 7]. Tcf-1-KO mice developed a lower number of memory CD8 T cells, which shows further defects in expansion during recall responses [7]. Analysis of the epigenetic map also differed between effector and memory CD8 T cells [9]. For example, members of the de novo DNA methyltransferase (DNMT) family such as DNMT1, DNMT3a or DNMT3b, regulate epigenetic reprogramming during effector and memory differentiation [10, 11].

On activated CD8 T cells, NKG2D was originally described as a co-stimulatory receptor, which enhances TCR-induced effector functions [12–14]. Recently, NKG2D signaling was also shown to induce the transcription of memory-associated genes, such as Eomes and CD62L, by weakly activating mTORC1 complex [15]. In addition, several studies showed a relationship between NKG2D and IL-15 signaling [16]. NKG2D signaling favors memory commitment of CD8 T cells by enhancing IL-15-mediated PI3K signaling [17]. We also showed that NKG2D signaling and memory formation are linked. Triggering NKG2D signaling during priming rescued the memory recall responses of deeply dysfunctional CD4-unhelped CD8 T cells [18]. However, another study suggested that NKG2D signaling has no major role in memory CD8 differentiation, but contributes by increasing the effector function of memory CD8 T cells upon recall responses [19]. In view of these controversial correlations between NKG2D and memory formation, the importance of NKG2D signaling in memory formation is still unclear.

In this study, we hypothesize that during killing by CD8 T cells, NKG2D signaling provides a certification that results in the selection of the best-suited CD8 T cells for differentiation into memory cells. To test this, we developed an experimental model in which every step of an immune response is temporally controlled, using both open (polyclonal) and closed (monoclonal) CD8 T cell repertoires. The closed repertoire consisted of transferring genetically marked pMel TCR-transgenic CD8 T cells into C57BL/6 wildtype hosts and concomitant priming with dendritic cells (DC) pulsed with the melanocytic human gp100 peptide (hgp100), their cognate antigen [20]. In our open repertoire model, the endogenous CD8 T cells were primed using the LCMV-derived viral epitope gp33 [21]. In both models, NKG2D signaling was temporarily blocked with an injection of a neutralizing antibody during the effector phase (6 days after priming). We found that NKG2D signaling during the effector phase was crucial for the development of functional memory CD8 T cells, as its blocking led to the formation of a functionally defective pool of memory cells. Interestingly, temporary blockade of NKG2D signaling did not alter the early response to IL-15 stimulation. However, it reduced the level of phosphorylated ribosomal protein S6 and changed the mRNA levels of epigenetic modifiers, such as DNMT3a and DNMT3b. Finally, the protective capacity of memory pMel CD8 T cells against B16 tumor challenge was greatly reduced. Our data suggest that NKG2D signaling during the effector phase regulates the translational machinery and epigenetic remodeling of effector CD8 T cells, with functional consequences that extend to the memory CD8 T cells. We propose a model of memory certification that occurs during the effector phase, in which CD8 T cells receiving NKG2D signaling in combination with TCR engagement will be certified to differentiate into functional memory cells.

## Materials and methods

### Mice and cells

All cells were cultured in RPMI supplemented with 10% heat-inactivated fetal bovine serum (Seradigm), 2 mM L-glutamine (Corning), and 1% penicillin/streptomycin (Corning), with the exception of melanoma B16 cells, which were cultured in similarly supplemented DMEM (Corning). All mice were housed at Loyola University Chicago in a pathogen-free facility. OT-I (C57BL/6-Tg[TcraTcrb]1100Mjb/J) Thy1.1^+^ and pMel (B6.Cg-Thy1a/CyTg[TcraTcrb]8Rest/J) Ly1.1^+^ TCR transgenic mice were bred in house. Eight- to twelve-week-old C57BL/6 were purchased from The Jackson Laboratory. Animal experiments were conducted in accordance with Loyola University Chicago Institutional Animal Care and Use Committee guidelines.

### DC generation and CD8 isolation for in vivo priming

To generate DC, bone marrow isolated from C57BL/6 mice were cultured in 6-well plates for 7 days in presence of GM-CSF. On day 6, DC were activated overnight by 1 μg/ml lipopolysaccharide (Sigma-Aldrich). Activated DC were pulsed at 10 × 10^6^ cells/ml with 10 μg/ml of hgp100 (KVPRNQDWL) or gp33 (KAVYNFATM) peptide for 2 h at room temperature (RT). DC (5 × 10^5^) were injected subcutaneously on each flank of C57BL/6 mice. In parallel, mice were injected retro-orbitally with 2.5 × 10^5^ pMel CD8 T cells isolated from spleens and magnetically purified using CD8a + T cell Isolation Kit (Miltenyi Biotech) according to the manufacturer’s protocol. Six days later half of the mice were intraperitoneally injected with 500 μg of anti-NKG2D antibody, clone HMG2D or hamster IgG (BioXCell) [22–26], as described in the figure legend.

### In vivo killing assay

OT-I Thy1.1^+^ splenocytes (10 × 10^6^ cells/ml) were divided into three groups and pulsed with 10 μg/ml of gp33 peptide, hgp100 peptide or kept unpulsed for 15 min at 37 °C in medium. After extensive washings, each group was loaded with CFSE (2 μM, 0.2 μM and 0.02 μM respectively) in PBS (without calcium and magnesium) for 10 min at 37 °C, followed by neutralization on cold medium for 5 min on ice. After washings, 15 × 10^6^ cells were injected retro-orbitally in immunized and control mice. Eighteen hours later, mice were euthanized, and spleens were harvested for flow cytometry analysis [27]. Injected target cells were identified by staining for the congenic marker CD45.1. Percent specific lysis = [1-(Non-transferred control ratio/Experimental ratio)] × 100. Results were normalized to responses in a naïve control mouse.

### Ex vivo CD8 T cell restimulation

Hgp100 or gp33 peptide (1 μg/ml) was added to 2 × 10^6^ splenocytes and incubated overnight at 37 °C in presence of Brefeldin A. To induce phosphorylation of STAT5, 5 × 10^5^ splenocytes were stimulated with 5 ng/ml recombinant murine IL-15 (Peprotech) for 30 min at 37 °C. Unstimulated cells were used as a control. The induction of pSTAT5 was stopped by direct addition of fixation buffer (Biolegend), followed by methanol fixation and permeabilization.

### Flow cytometry

Fluorochrome-conjugated antibodies against CD3, CD8, CD44, CD62L, CD69, Tim-3, PD-1, CD90.1, CD45.1, Granzyme B, Tbet, Bcl-2, IFN-γ, IL-2 (Biolegend), NKG2D, CD25, TNF-α, KLRG1 (eBioscience), pSTAT5 (Invitrogen), CD127 (BD Bioscience) and pS6 (Cell Signaling Technologies) were used. Cell surface staining, intracellular staining, and flow cytometry analysis was performed as previously described [28]. Staining of phosphorylated proteins was performed following eBioscience protocol with methanol fixation and permeabilization. For analyses, cells were gated on live cells using Zombie Aqua exclusion dye (Biolegend). pMel cells were distinguished from endogenous cells by gating on the congenic marker CD90.1 and in vivo CTL assay analyses were performed after gating on the congenic marker CD45.1. When methanol was used, live cells were defined based on size.

### mRNA isolation and real-time PCR

pMel CD8 T cells were isolated from the spleen of 5 pooled mice using FACS cell sorter. After purification with RNeasy Protect Kit (Qiagen), 10 ng of mRNA was used as a template for cDNA using RT^2^ PreAMP cDNA Synthesis Kit (Qiagen). cDNA was subsequently pre-amplified by PCR using 84 different sets of primers, corresponding to RT^2^ Profiler™ PCR Array Mouse Epigenetic Chromatin Modification Enzymes (Qiagen). Real-time PCR was run on QuantStudio 6 Flex (Applied Biosystems) using RT^2^ SYBR Green ROX qPCR Mastermix (Qiagen).

### Tumor challenge

Tumor challenge experiments were performed by intradermally injecting 1 × 10^5^ B16 tumor cells. Four to five mice were used per group. Tumor size was measured using a caliper every 2–3 days and tumor area was calculated using the following formula: (π*length*width)/4.

### Statistic

Cells from individual tissues from 5 mice per group were analyzed for statistical significance using a two-tailed Student’s t test in order to obtain a 5% significance level with a 95% CI. The number of mice used per experiment was validated using StatMate2 (GraphPad Software, Inc.). For memory response analyses, mice with pMel frequency close to detection limit were excluded (1–2 mice / group on average). Differences in anti-tumor responses were considered statistically significant at *p* value of < 0.05, using a 2-way ANOVA test with Bonferroni correction for multiple comparisons. Tumor-free survival was plotted by Kaplan-Meier plots and compared by log-rank analysis.

## Results

### Temporary NKG2D blockade during effector phase results in the formation of non-cytolytic memory CD8 T cells

**Fig. 1:**
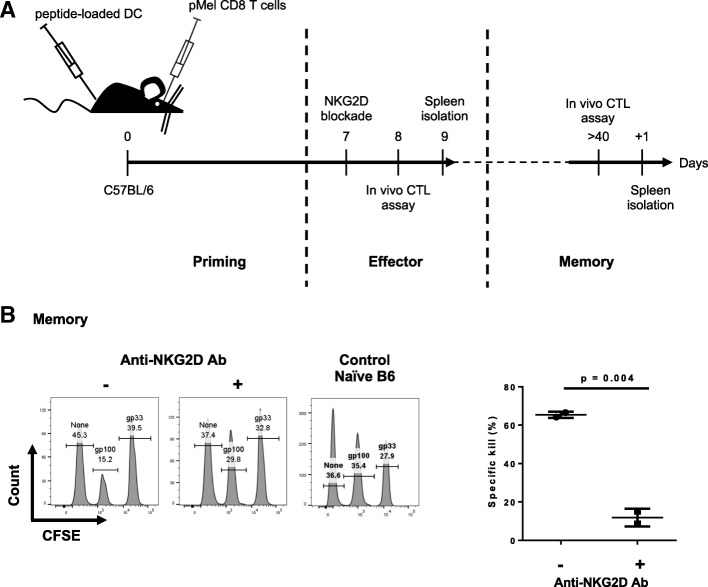
NKG2D blockade during effector phase resulted in the formation of non-cytolytic memory CD8 T cells. **a** Schematic representation of the experimental design used to block NKG2D during the effector phase. At day 0, mice were immunized with peptide-loaded DC subcutaneously and injected retro-orbitally with purified pMel CD8 T cells. One week after immunization, half of the mice were injected intra-peritoneal with the anti-NKG2D blocking antibody (Ab) a day prior to the in vivo CTL assay. This period corresponds to the effector phase. Memory recall responses were analyzed at least one month later by repeating the in vivo killing assay. **b** Example of the in vivo killing assay readout by flow cytometry during memory responses. Immunized mice were injected with three populations of target splenocytes, each loaded with different amounts of CFSE and pulsed with different peptides. Spleens were analyzed 18 h later by flow cytometry and the ratios between the peptide-pulsed population vs. the unpulsed population were calculated and normalized to the naïve control mouse shown in the figure. The quantification of specific killing is summarized in the graph. Data shown is representative of four independent experiments

To analyze the contribution of NKG2D signaling in the formation of memory CD8 T cells, we developed an experimental mouse model where NKG2D was transiently blocked. C57BL/6 mice were injected with purified CD8 T cells isolated from pMel mice. Concurrently, mice were immunized with activated hgp100-pulsed DC (Fig. 1a). NKG2D signaling was blocked in vivo with a single injection of an anti-NKG2D blocking antibody at day 6, followed by an injection of peptide-loaded target cells. Expression in target cells (proceeded splenocytes) of NKG2D ligand was corroborated by flow cytometry (Additional file 1). HMG2D specificity for NKG2D was tested by using hamster IgG control (Additional file 2).

Thus, effector CD8 T cells interacted with their target in presence or absence of NKG2D signaling. The functionality of memory CD8 T cells generated under these conditions was assessed at least one month later by performing an in vivo CTL killing assay (in vivo CTL). Memory pMel cells were able to kill more than 60% of their target cells (Fig. 1b). However, if these memory CD8 T cells did not engage NKG2D at the effector phase, their killing capacity was strongly reduced during memory recall responses with only 12% of the target cells killed (Fig. 1b). These results suggest that NKG2D signaling was important during the effector phase for the formation of cytolytic memory CD8 T cells. We confirmed that the anti-NKG2D antibody was indeed blocking (as previously described [29]) and not depleting by detecting the presence of pMel cells in the spleen one or eight days after antibody injection and comparing to untreated mice (Additional file 3). In addition, memory CD8 T cells expressed NKG2D on their cell surface, confirming that the blockade was indeed transient (Additional file 4).

### NKG2D blockade during effector phase leads to the generation of defective cytokine responses by memory CD8 T cells

**Fig. 2:**
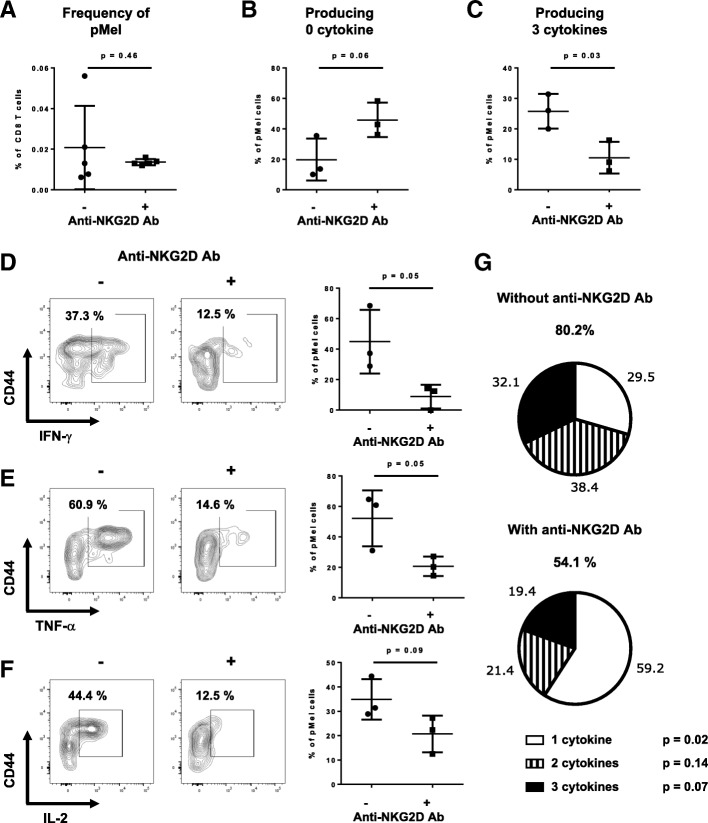
NKG2D blockade during effector phase led to the generation of defective cytokine responses by memory cells. Memory pMel CD8 T cells were generated as in Fig. 1a. **a** Representative graph shows the percentage of memory pMel cells (CD90.1^+^) among total CD8 T cells present in the spleen one day after the in vivo killing assay. **b**-**g** Splenocytes from (A) were restimulated overnight with hgp100 peptide or gp33 (irrelevant peptide). Cytokine production by CD90.1^+^ pMel cells was measured the next day by flow cytometry. The percentage of memory pMel CD8 T cells that produce 0 (**b**) or 3 cytokines (**c**) is shown. **d**-**f** Shown are flow examples and graphs summarizing the percentage of pMel CD8 T cells secreting IFN-γ (**d**), TNF-α (**e**) or IL-2 (**f**). **g** Pie charts show the percentage of pMel cells producing 1, 2, or 3 cytokines, among the cells that produce at least one cytokine (denoted above each pie chart). For (**b**-**g**), 2 mice/group were excluded due to too low frequency of recovered pMel cells. Data shown are representative of three independent experiments

Memory pMel CD8 T cells lacking NKG2D signaling during effector phase were unable to effectively kill their targets in vivo (Fig. 1b). It is possible that in absence of NKG2D signaling pMel CD8 T cells did not survive at a frequency allowing an efficient memory recall response. This was excluded, as the percentages of memory pMel cells generated with or without NKG2D blockade were not significantly different, despite a tendency of recovering slightly less pMel cells in the NKG2D-blocked group (Fig. 2a).

We then determined the capacity of these cells to produce cytokines (IFN-γ, TNF-α and IL-2) ex vivo. The proportion of non-cytokine-producing memory cells increased from 19.8 to 45.9% when NKG2D signaling was blocked (Fig. 2b), while the proportion of triple cytokine producing cells (one major criteria defining functional memory cells [30, 31]) decreased from 25.8 to 10.5% (Fig. 2c). The production of each cytokine was reduced (Fig. 2d-f), confirming that the decrease in cytokine production was affecting all 3 cytokines and was not restricted to only one of them. The cytokine distribution within the cytokine-producing pMel cells was altered. The majority of these cells produced only one cytokine, as shown by the increase from 29.5 to 59.2%. In contrast, most memory pMel CD8 T cells formed in the presence of NKG2D signaling produced 2–3 different cytokines (Fig. 2g).

**Fig. 3:**
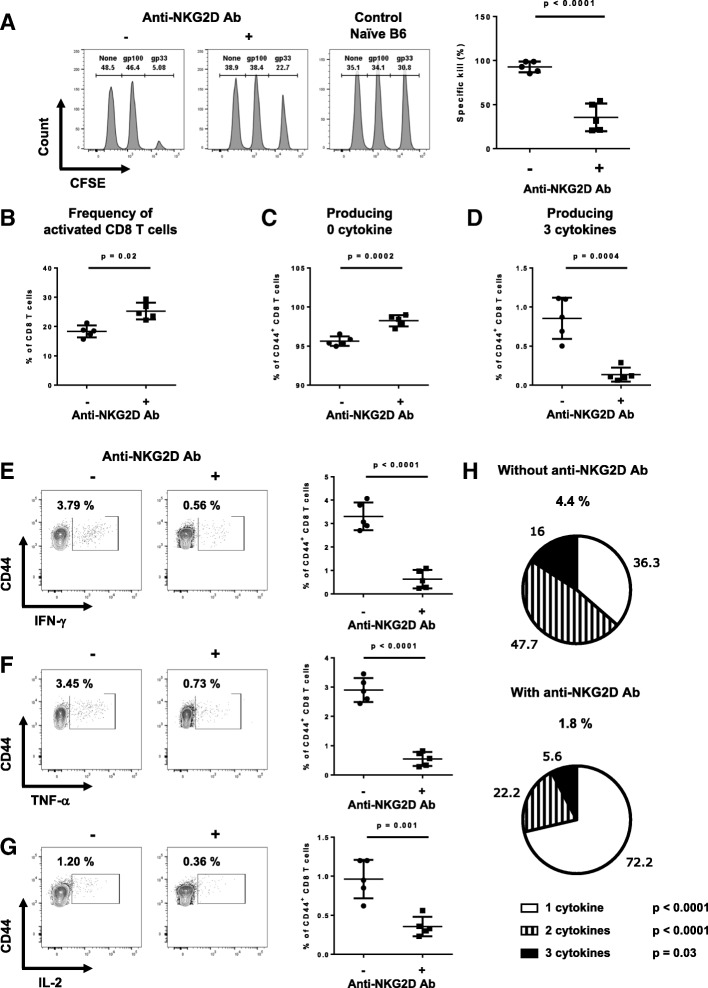
Blocking NKG2D during effector responses of endogenous CD8 T cells resulted in highly defective memory responses (**a**) Example of the in vivo killing assay readout by flow cytometry during memory responses. **b** Graph shows the percentage of antigen-experienced (CD44^+^) CD8 T cells among total CD8 population present in the spleen one day after the in vivo killing assay. **c**-**h** Splenocytes from (**b**) were restimulated overnight with gp33 peptide or irrelevant peptide (hgp100 peptide). Cytokine production was measured the next day by flow cytometry. The percentages of endogenous CD44^+^ CD8 T cells that produce 0 (**c**) or 3 cytokines (**d**) are shown. **e**-**g** Shown are flow examples and graphs summarizing the percentage of endogenous CD44^+^ CD8 T cells secreting IFN-γ (**e**), TNF-α (**f**) or IL-2 (**g**). **h** Pie charts show the percentage of endogenous CD44^+^ CD8 T cells secreting 1, 2, or 3 cytokines among the cells that produce at least one cytokine (denoted above each pie chart). Data shown are representative of two independent experiments

These results were confirmed in our open repertoire model. Endogenous memory CD8 T cells that were formed in absence of NKG2D signaling were strongly impaired in their ability to kill gp33-loaded target cells in vivo as the blocked cells showed only 25% of specific killing vs. 95% in non-blocked cells (Fig. 3a), despite a similar percentage of activated CD8 T cells (Fig. 3b). Ex vivo, NKG2D-blocked cells produced little to no cytokines (Fig. 3c-g). The cytokine-producing cells also displayed a significant loss of polycytokine production (Fig. 3h).

The defect in cytolytic function and cytokine production could not be linked to a specific memory phenotype as there were no changes in the expression of CD62L, CD44, KLRG1, T-bet or Bcl-2 in cells that had NKG2D signaling blocked. Similar expression of PD-1, Tim-3 and granzyme B excluded a difference in activation or exhaustion phenotype (Additional file 4). Altogether, these data showed that the absence of NKG2D signaling led to the generation of profoundly dysfunctional memory cells with impaired cytolytic capacity and polycytokine production.

### NKG2D blockade minimally affects the effector CD8 T cell responses

**Fig. 4:**
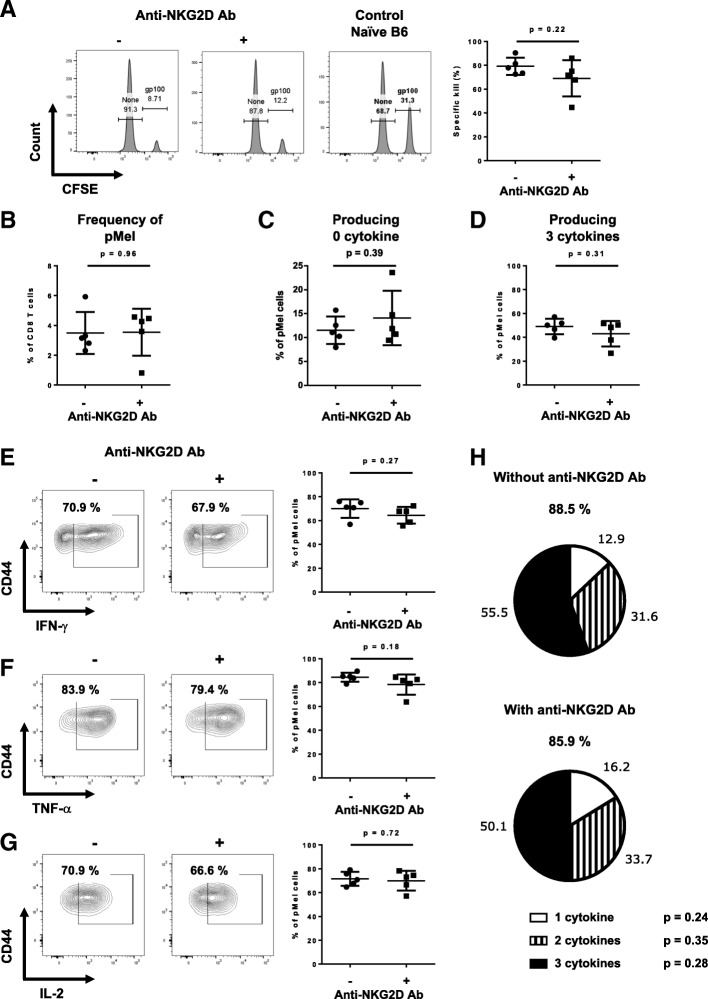
NKG2D signaling is not needed on activated pMel CD8 T cells for proper effector functions. Effector pMel CD8 T cells were generated as described in Fig. 1a. One day before the in vivo killing assay, half of the mice were injected with the anti-NKG2D blocking antibody. **a** Example of the in vivo killing assay readout by flow cytometry during memory responses. **b** Graph shows the percentage of effector pMel CD8 T cells (CD90.1^+^) among total CD8 T cells present in the spleen one day after the in vivo killing assay. **c-h** Splenocytes from (**b**) were restimulated overnight with hgp100 peptide or gp33 peptide. Cytokine production was measured the next day by flow cytometry. The percentages of effector pMel CD8 T cells (CD90.1^+^) that produce 0 (**c**) or 3 cytokines (**d**) are shown. **e**-**g** Shown are flow examples and graphs summarizing the percentage of pMel CD8 T cells secreting IFN-γ (**e**), TNF-α (**f**) or IL-2 (**g**). **h** Pie charts show the percentage of pMel cells producing 1, 2, or 3 cytokines, among the cells that produce at least one cytokine (denoted above each pie chart). Data shown are representative of five independent experiments

It is plausible that the defects seen in the memory recall responses were the result of poor effector responses. We therefore tested the in vivo cytolytic capacity of effector pMel cells one day after injection of NKG2D-blocking antibody. No significant differences were observed in their ability to kill their targets when compared to the non-blocked pMel CD8 T cells (Fig. 4a). The percentage of pMel CD8 T cells in the spleen was also similar between the two groups (Fig. 4b). The percentage of cytokine-producing cells as well as the quality of the polycytokine production were not significantly impacted by blocking NKG2D signaling (Fig. 4c-g), nor did the cells present any different activation phenotype (Additional file 4). We obtained similar results in the polyclonal model. The lack of NKG2D staining measured by flow cytometry in the treated mice confirmed the binding specificity of the blocking antibody (Additional file 5). Gp33-primed endogenous CD8 T cells showed a small, but not significant decrease in cytolytic function and a small decrease in cytokine production by endogenous NKG2D-blocked CD8 T cell (Additional file 6). This decrease in cytokine production is conformed to the canonical role of NKG2D on activated CD8 T cells, which consists in enhancing cytokine production. Altogether, these data showed that blocking NKG2D signaling had minimal effect on the effector responses of pMel CD8 T cells, while it resulted in strong impairment in memory responses.

### Blocking NKG2D in vivo did not influence early IL-15 response of effector CD8 T cells

**Fig. 5:**
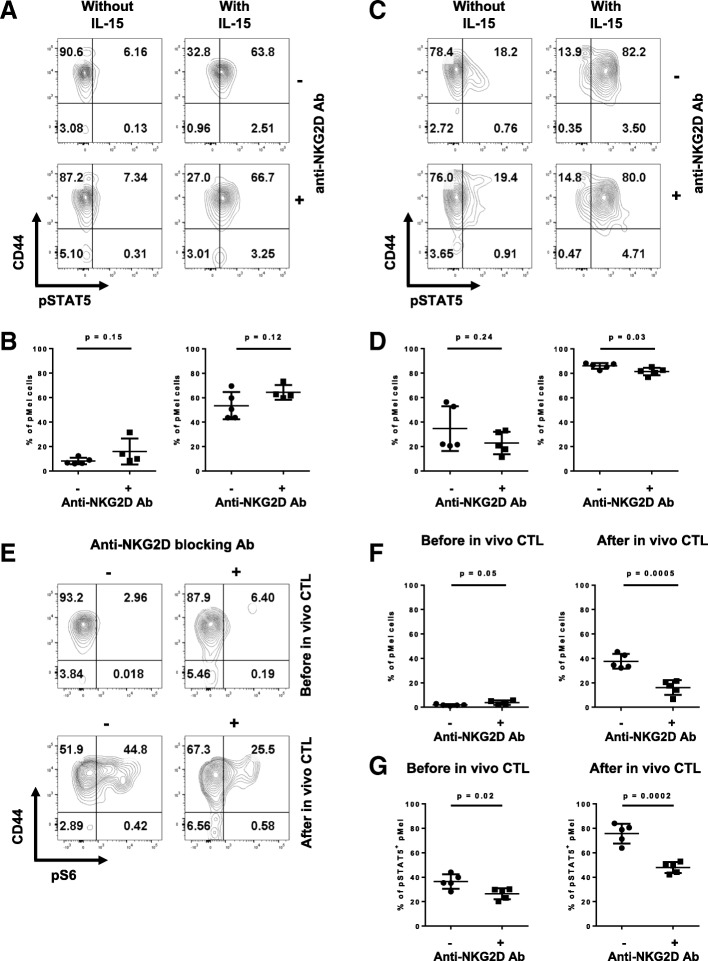
Early responses to IL-15 was not affected by NKG2D blockade, while S6 phosphorylation was reduced. Effector pMel CD8 T cells were generated as described in Fig. 1a. Shown are the representative plots (**a**, **c**) of pSTAT5 staining and graphs (**b**, **d**) summarizing the percentage of pMel CD8 T cells (CD90.1^+^) expressing pSTAT5 after ex vivo exposure to IL-15 for 30 min. Splenocytes were isolated one day after injecting the anti-NKG2D blocking antibody (**a**, **b**) or one day after the in vivo killing assay (**c**, **d**). Unstimulated cells were used as control. Representative plots (**e**) and graphs (**f**) summarizing the percentage of pMel cells (CD90.1^+^) expressing pS6 ex vivo before and after the in vivo killing assay in the presence or absence of NKG2D blockade. **g** Splenocytes from (EF) were stimulated with IL-15 for 30 min. The percentage of pSTAT5^+^ pMel cells that express pS6 is summarized in the graph. Data shown are representative of two independent experiments

IL-15 is a key component in the survival of memory precursor CD8 T cells [4, 5]. NK cells lacking the adaptor molecule DAP10, which is required for NKG2D signaling [32], fail to respond to IL-15 stimulation [33]. Based on this association between IL-15 and NKG2D, we sought to determine if effector CD8 T cells undergoing temporary NKG2D blockade might be less responsive to IL-15, consequently impairing their differentiation into functional memory cells. We therefore assessed the IL-15-responsiveness of pMel cells by measuring the phosphorylation of STAT5 upon ex vivo IL-15 stimulation one day after NKG2D blockade (prior to the in vivo CTL assay). IL-15 responsiveness was not diminished after NKG2D blockade as phosphorylated STAT5 was detected in 60% of the pMel cells, similar to their non-blocked counterparts (Fig. 5a-b).

We then tested if target cell recognition by CD8 T cells influence their response to IL-15. We repeated the experiment described above, but measured IL-15 responsiveness one day after the in vivo CTL assay. Ex vivo exposure to IL-15 induced phosphorylation of STAT5 in 86% of the cells, but no significant differences in the level of phosphorylation was observed between the two groups (Fig. 5c-d). We also tested phosphorylation of STAT5 in response to IL-15 in a time course. We found no differences in the levels of STAT-5 phosphorylation in pMEL that received NKG2D blockade or not (Additional file 7). Thus, the functional defects induced by the lack of NKG2D signaling during the in vivo killing phase cannot be explained by a decrease in the pMEL responses to IL-15.

### NKG2D blockade in effector CD8 T cells results in reduced S6 phosphorylation

Several studies have demonstrated the role of mTORC1 in the decision of T cell fate. Low mTORC1 activity is associated with memory cell differentiation, while high mTORC1 activity is associated with terminally-differentiated effector cells [15, 34]. Phosphorylation of the ribosomal protein S6, a downstream target of mTORC1, can be used as a readout of mTORC1 activity [35]. As both NKG2D and TCR signaling activate the mTORC1 pathway, we investigated the consequences of NKG2D blockade on S6 phosphorylation by measuring phosphorylated S6 (pS6) in effector CD8 T cells prior to the in vivo CTL assay and one day afterwards. Target killing was necessary to induce pS6 in vivo, as we found no phosphorylation prior to the in vivo CTL assay. As NKG2D contributes to memory formation, we expected to find higher pS6 upon NKG2D blockade. However, NKG2D blockade decreased the percentage of pS6 by half, from 44.8 to 25.5% (Fig. 5e-f).

We showed that early IL-15 signaling was not affected by NKG2D blockade. As cytokines have been reported to induce S6 phosphorylation [36], we assessed the level of pS6 in pMel CD8 T cells that responded to IL-15 stimulation (pSTAT5^+^ pMel cells). In absence of in vivo killing (in vivo CTL assay), 26.5% of NKG2D-blocked pMel T cells and 36.5% of their non-blocked counterparts were pS6^+^. Although significant, this difference was small (Fig. 5g). After the in vivo CTL assay, pMel CD8 T cells were more efficient at phosphorylating S6, as 75.7% of them were pS6^+^. Upon NKG2D blockade, only 47.9% of pMel cells phosphorylated S6 (Fig. 5g). Altogether, these data showed that blocking NKG2D in vivo results in a general reduction in the capacity of effector CD8 T cells to phosphorylate S6.

### NKG2D signaling alters the expression of epigenetic modifiers in CD8 T cells

**Fig. 6:**
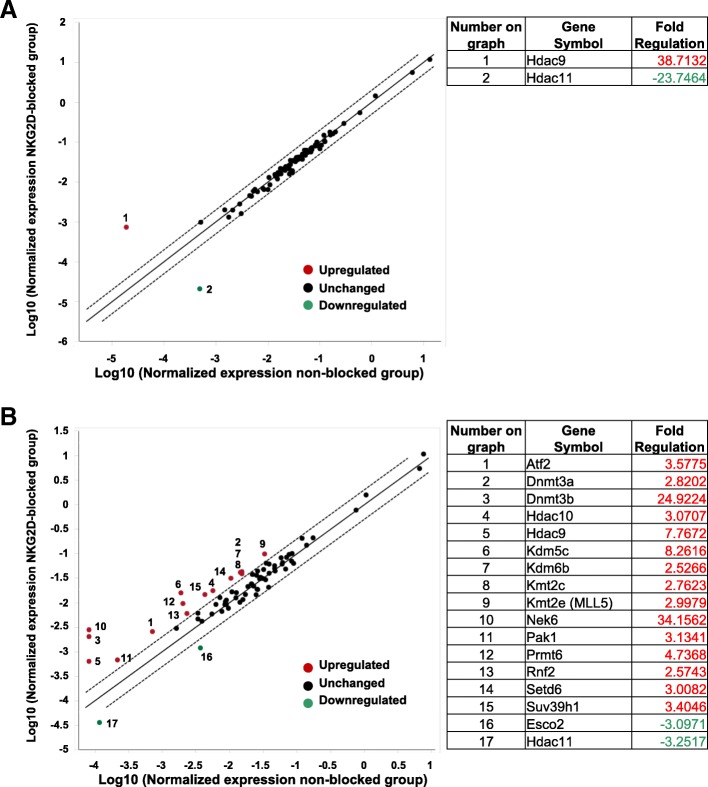
Blocking NKG2D signaling during in vivo killing altered the expression of epigenetic modifier enzymes. Effector pMel CD8 T cells were generated as described in Fig. 1a. One day after injecting the anti-NKG2D blocking antibody (**a**) or one day after the in vivo killing assay (**b**), effector CD8 pMel T cells were isolated from the spleen of 5 pooled mice using CD90.1^+^ congenic marker. After mRNA isolation and conversion into cDNA, 84 different epigenetic modifier enzymes were selectively quantified using RT2 Profiler Epigenetic modifier enzymes PCR array. The fold of change was calculated using the online software provided by Qiagen. The enzymes with > 2.5-fold change in expression are numbered on the plot and summarized in the tables on the right side, together with their fold of change

The defect observed in memory CD8 T cells formed upon NKG2D blockade could not be attributed to a quantitative defect (reduced number of memory cells), nor a reduced ability to respond to IL-15. Only the quality of the memory cells was impacted, suggesting epigenetic reprogramming by NKG2D signaling. To test this, we isolated mRNA from purified effector pMel CD8 T cells before and after the in vivo CTL assay in the presence or absence of NKG2D blockade. We analyzed the transcripts of 84 different epigenetic modifier enzymes. Prior to the in vivo CTL assay, NKG2D blockade induced minimal changes in mRNA levels, with only two enzymes displaying altered expression (Fig. 6a). However, target killing in the absence of NKG2D signaling resulted in changes in expression of 17 epigenetic modifier enzymes (Fig. 6b). Two of these transcripts were found to be highly upregulated: Nek6 (34.2 fold increase) and DNMT3b (24.9 fold increase). The function of Nek6 has not yet been reported in CD8 T cells. DNMT3b, together with DNMT3a (which is 2.8 fold upregulated), belong to the only family known to de novo add methyl groups on DNA [37]. While DNMT3b function in T cell differentiation is unclear, DNMT3a has been shown to control the expression of genes associated with T cell memory formation [10, 38]. These data indicate that NKG2D signaling regulates the expression of epigenetic modifiers in effector CD8 T cells, which could result in aberrant DNA methylation leading to the development of functionally defective memory CD8 T cell.

### NKG2D blockade during the formation of memory CD8 T cells impairs their ability to protect against tumor

**Fig. 7:**
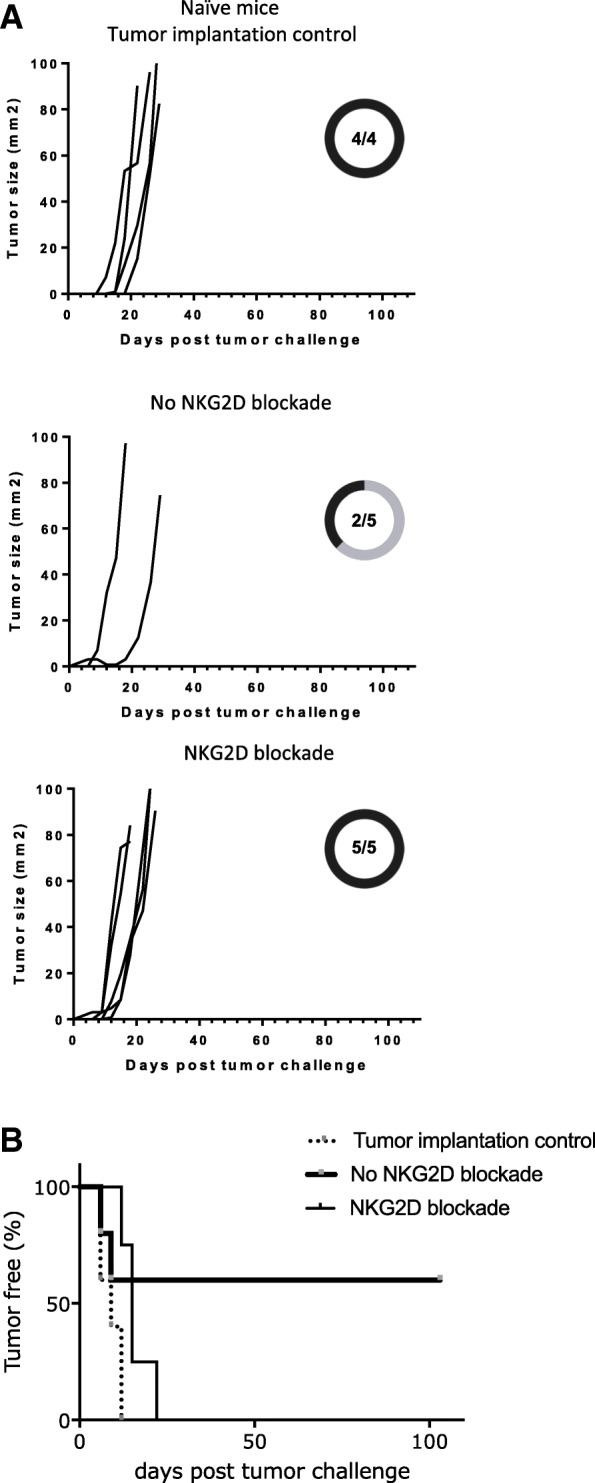
Memory cells formed upon transient NKG2D blockade were not protective against melanoma B16 tumor. Memory pMel CD8 T cells were generated as in Fig. 1a. (**a**-**b**) B16 melanoma cells were injected during the memory phase (>d40 after the in vivo CTL assay and NKG2D blockade). Tumor growth (**a**) and incidence (**b**) were followed over time. Individual lines in graphs shown in A represent the tumor growth of a single mouse. Tumor incidence in mice with memory pMel CD8 T cells formed in absence (top graph) or presence (middle graph) of transient NKG2D blockade is indicated on each graph. Naïve C57BL/6 mice were used as control. Proportion of tumor take are also represented. The graph shows the results of one of two independent experiments

Finally, we tested the protective capacity of memory pMel CD8 T cells formed during transient NKG2D blockade. At memory phase, mice were challenged with B16 melanoma cells instead of the in vivo CTL assay. Tumor incidence and growth kinetics were monitored over time and compared to mice in which memory pMel CD8 T cells were formed in presence of NKG2D signaling. Without NKG2D blockade, tumor protection was achieved in 60% of the mice (Fig. 7a, top graph). However, 0% of the mice were protected by memory cells formed during transient NKG2D blockade (Fig. 7a, middle graph) and their tumor growth kinetic was comparable to naive C57BL/6 mice used as a control (Fig. 7a, bottom graph and Fig. 7b). Similar trends were observed in a second experiment (Additional file 8). These data demonstrate that the absence of NKG2D signaling during the effector phase results in non-protective memory CD8 T cells.

## Discussion

In this study, we showed that NKG2D signaling in CD8 T cells is necessary during the effector phase for the development of functional memory cells. We call this essential step “memory certification”, a process of molecular accreditation that is received during the effector phase-killing phase when CD8 T cells engage their targets. In absence of this certification, memory CD8 T cells are formed, but display defective recall characteristics, as demonstrated by their inability to kill target cells and impaired polycytokine production. These defects observed in uncertified CD8 T cells were not attributable to lower numbers of memory precursor cells, cell phenotype, expression of inhibitory receptors, or cytokine unresponsiveness. Importantly, we found that in these cells S6 phosphorylation was reduced and that the content of epigenetic modifiers was also altered. Thus, during the effector phase, NKG2D signaling provides CD8 T cells with the appropriate program to become functional memory cells.

The presence and ability to respond to IL-15 is crucial for the survival and homeostasis of memory and memory precursor T cells [4, 5]. Several studies have shown that NKG2D and IL-15 receptor signaling are coupled [16]. For example, Horng et al. genetically modified NK cells to abrogate NKG2D expression by forcing DAP10 ubiquitination and degradation. In absence of DAP10, NK cells were unable to respond to IL-15 stimulation despite expressing similar levels of IL-15 receptors as wildtype NK cells. They further demonstrated that DAP10 co-immunoprecipitated with the IL-15 receptor complex, suggesting a physical interaction between DAP10 and IL-15 receptor complex in NK cells. While DAP10 was required for IL-15-induced signaling in NK cells, in CD8 T cells, it was dispensable for IL-15-induced signaling [16]. Using in vitro activated wildtype and NKG2D-KO OT-I CD8 T cells, Wensveen et al. showed that phosphorylation of STAT5 upon IL-15 stimulation was not affected by the absence of NKG2D. However, PI3K signaling was reduced which correlated with lower accumulation of Mcl-1, a pro-survival molecule [17]. In their in vivo model, NKG2D deficiency significantly reduced the formation of central memory precursors. The authors concluded that NKG2D was not necessary for recall memory responses but was important for the survival of central memory precursors. This is in sharp contrast with our results, as we showed that NKG2D signaling during the effector phase shaped the quality of the memory being formed, without altering the number or phenotype of memory cells. The different mouse models used to investigate the importance of NKG2D on memory formation might explain the discrepancy between our two studies. In their model, wildtype and NKG2D-KO T cells were mixed at a 1:1 ratio prior to injection. While this model has the advantage of comparing wildtype and NKG2D-KO CD8 T cells under the same conditions in the same host, these cells are also competing for the killing of target cells and access to survival factors (for example IL-2). In such competing conditions, we expect that cells expressing NKG2D will have an advantage, as the canonical role of NKG2D is to enhance T cell functions and survival. Additionally, these experiments did not test if T cells lacking NKG2D could kill and survive under non-competing condition. The use of NKG2D-KO CD8 T cells prohibits the separation of NKG2D functions during the effector and the memory phase. To overcome this problem, we injected NKG2D blocking antibody in vivo once at the effector phase, just prior to the in vivo CTL assay. This allowed us to determine if NKG2D signaling during the effector phase is necessary for memory formation without compromising NKG2D signaling at the memory phase.

CD4 T cells are another important modulator in the formation of protective memory. Studies including ours have shown that CD8 T cell priming in absence of CD4 T cell help resulted in functionally defective effector CD8 T cells and the development of subsequent defective memory T cells [18]. We showed that providing NKG2D ligands during immunization in absence of CD4 T cells restored the protective memory response of these CD4-unhelped CD8 T cells despite providing no improvement to the effector CD8 T cell response [18]. In our current model, CD4 T cells were present during priming. The peptide-pulsed DC used in this study were in vitro matured with LPS prior injection. This bacteria-derived protein induces MHC I and II upregulation, as well as the expression of various co-stimulatory molecules on DC, which can mediate both CD4 and CD8 T cell priming. Under such conditions, we can assume that pMel CD8 T cells received CD4 T cell help during priming in the LN and that CD8 T cell priming occurred under optimal conditions prior to blocking NKG2D signaling. In humans, a rare CD4 T cell population has been reported to express NKG2D [39]. Such a population has not been described in mice so far. It is unlikely that the injected anti-NKG2D blocking antibody directly targeted CD4 T cells at the effector phase. Consequently, it is safe to assume that in our experiment design, only activated CD8 T cells were impacted by the blockade, and only transiently.

Ribosomal protein S6, a component of the 40S ribosomal subunit, is a downstream target of multiple pathways, such as TCR, co-stimulatory and nutrient signaling [40]. Of the few kinases known to phosphorylate S6, S6K1 has been extensively investigated in relation to mTORC1 signaling. S6K1 is one of the main downstream targets of mTORC1, an important complex involved in the control of memory vs. effector CD8 T cell differentiation [41]. In our model, we showed that in absence of NKG2D signaling the level of pS6 was reduced, which would imply lower mTORC1 activity and more memory cells. However, we did not observe any difference in total memory cell number formed under transient NKG2D signaling blockade conditions, suggesting that pS6 controls memory formation in an mTORC1 independent pathway. This possibility is further supported by the study from Salmond et al. showing that T cell activation is influenced by mTORC1 / S6K1 pathway independently of S6 [42]. Using a mouse model in which all five phosphorylation sites of S6 were mutated, Ruvinsky et al. showed a reduction in the translation efficiency of few specific mRNA. Some of them were related to energy consumption, which resulted in reduced sources of ATP in muscle cells [40]. It is, therefore, possible that the lower level of pS6 observed in uncertified CD8 T cells altered their translational machinery as well as the abundance of readily available energy sources. In addition to NKG2D and TCR, growth factors and cytokines have been reported to induce S6 phosphorylation [36]. Similar to TCR engagement, IL-15 stimulation also resulted in lower S6 phosphorylation upon NKG2D blockade, even though the early IL-15 response was not affected by the blockade. These data imply that IL-15 cannot compensate for the lack of NKG2D signaling. Our data also suggest that NKG2D blockade compromised S6 phosphorylation independently of the source of the stimulation, indicating that S6 is an important mediator downstream NKG2D signaling.

RT-PCR results on epigenetic modifier enzymes suggested that NKG2D signaling downregulates the expression of DNMT3a and DNMT3b, the two de novo DNA methyltransferase responsible for silencing the promoter region of various memory-associated genes such as TCF-1 [38]. Changes in the epigenetic profile are among the most profound modifications induced by changes in environmental signals [43, 44]. It is remarkable that the temporary blockade of only one environmental sensor (NKG2D) was sufficient to modify the expression pattern of multiple epigenetic modifier enzymes, with defective memory CD8 T cells as the ultimate outcome. Our data support the idea that the program acquired by CD8 T cells during the effector phase is key for their development into functionally capable memory cells and that this program/code is “bugged” by the absence of NKG2D signaling. Why is this defect not reversible since NKG2D is only temporarily blocked? This can be explained in part by the usually stable nature of epigenetic changes [45]. Another explanation could be articulated by the example of CD4 T cell commitment. During the differentiation of peripheral CD4 T cells, depending on the signals received during priming, these cells differentiate into for example Th1/Th2/Th17 subtypes/lineages; however, once one path is taken, switching among lineages is precluded [46].

The killing and clearance of target cells by effector T cells represent the initiating cues for the contraction phase and differentiation into memory cells [47]. These cues imply a tight control on cell cycle signaling, by either allowing the cells to enter division or by forcing them into quiescence. Disruption of NKG2D signaling resulted in an imbalance in the mRNA expression of various enzymes involved in controlling cell cycle. We observed upregulation of Suv39H1 mRNA levels, described to induce cell cycle arrest [48]. However, we also observed upregulation of PRMT6, KMT2e, and SetD6 described to promote cell cycle [49–51]. Despite these changes, the total number of uncertified CD8 T cells that differentiated into memory cells did not change. This altered control in cell cycle observed in uncertified CD8 T cells might prevent the progression of the best-fitted cells into memory differentiation. Additional functions were reported for some of these epigenetic modifiers, with potential implication in T cell functions. In Th2 cells, Suv39H1 has been reported to silence Th1-related genes, thus contributing to T cell lineage commitment and plasticity [45]. Moreover, SETD6 has been shown to methylate the NK-kB subunit RelA. The repression of NF-kB target genes resulted in reduced inflammatory responses by primary immune cells [52].

NKG2D ligands are strongly upregulated during cell stress responses, such as DNA damage, upon viral infection, or in tumor cells [53]. While viral infection are usually cleared by CD8 T cells, leading to the formation of protective memory cells, tumors escape control from the endogenous CD8 T cell [54, 55]. The presence of high levels of immune suppressive cytokines, such as TGF-β, combined with the lack of nutrients and oxygen negatively impact T cell effector functions by, for example, reducing TCR signaling and downregulating the expression of NKG2D [56–58]. Furthermore, some tumors were found to secrete soluble NKG2D ligands that act as a decoy mechanism, also resulting in the downregulation of NKG2D [59]. Under these conditions, activated tumor-reactive T cells would be killing in absence of NKG2D signaling, with limited long-term efficacy. Indeed, our data suggest that killing in absence of NKG2D signaling lead to the differentiation of aberrant memory T cells with reduced protective capacity against tumors.

## Conclusion

Our experiments were designed and conducted with the purpose of dissecting the role of NKG2D signaling and eliminating the confounding contribution of inflammation. It remains to be determined if all CD8 T cells must obtain NKG2D certification to become memory cells, as evolution is characterized by the use of redundant mechanisms. It is clear however, that under the conditions used in our study, NKG2D plays a fundamental role in CD8 T cell memory formation. Understanding how long-term memory cells are selected from the initial effector pool of CD8 T cells is an essential objective in immunology. It is evolutionarily advantageous for the adaptive immune system to populate the memory compartment with cells that have demonstrated the capacity to destroy their targets. We propose that the selection of experienced CD8 T cells requires a molecular accreditation that is only received if they have identified and killed the correct target. We provide evidence of a process of certification that could contribute to the development of better vaccines and adoptive cell therapies.

## Additional files

Additional file 1: Target cells used for the in vivo CTL assay express NKG2D ligands. (PDF 111 kb)

Additional file 2: HMG2D Ab is specific for NKG2D. (PDF 110 kb)

Additional file 3: The anti-NKG2D antibody clone HMG2D did not deplete NKG2D expressing cells. (PDF 120 kb)

Additional file 4: The absence of NKG2D during the effector phase did not alter the phenotype of the memory CD8 T cells. (PDF 120 kb)

Additional file 5: Blocking NKG2D did not alter the phenotype of effector CD8 T cells (DOCX 88 kb)

Additional file 6: Endogenous effector CD8 T cells have a slightly decreased effector response in the absence of NKG2D signaling. (PDF 157 kb)

Additional file 7: Time dependent phosphorylation of STAT-5 by IL15 is not affected by NKG2D blockade. (DOCX 49 kb)

Additional file 8: Memory cells formed upon transient NKG2D blockade were not protective against melanoma B16 tumor. (PDF 118 kb)

## Abbreviations

DC: Dendritic cells
DNMT: De novo DNA methyltransferase
hgp100: Human gp100 peptide
IL: Interleukin
In vivo CTL killing assay: In vivo CTL assay
NKG2D: Natural Killer Group 2D
pS6: phosphorylated S6
pSTAT5: phosphorylated STAT5
RT: Room temperature
T-bet: T-box transcription factor
Tcf-1: T cell factor-1
