# Supplementary material for: NKG2D signaling certifies effector CD8 T cells for memory formation
Source: J Immunother Cancer. 2019 Feb 18;7:48. doi: 10.1186/s40425-019-0531-2 (PMC6380053; doi:10.1186/s40425-019-0531-2)
Supplement: Supplementary file 1 — Target cells used for the in vivo CTL assay express NKG2D ligands. (PDF 111 kb) [file 40425_2019_531_MOESM1_ESM.pdf]

Additional File 1

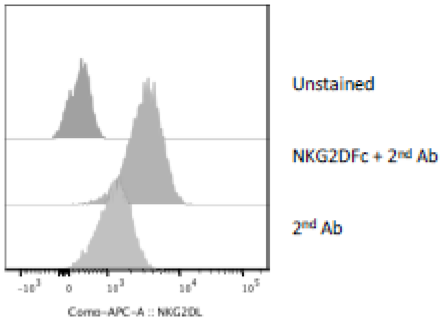

Additional File 1: Target cells used for the in vivo CTL assay express NKG2D ligands. Spleens were harvested from euthanized mice and prepared for use in the in vivo CTL assay. Briefly, spleens were subjected to ammonium chloride-based osmotic shock for red blood cell lysis, peptide loading, CFSE labelling, and adoptive transfer into a host mouse. Twelve hours after transfer, spleens were recovered from host mice. Expression of NKG2D ligands was confirmed by flow cytometry using NKG2D-Fc chimera in CFSE + cells. Unstained and secondary antibody alone are shown.
