# Supplementary material for: NKG2D signaling certifies effector CD8 T cells for memory formation
Source: J Immunother Cancer. 2019 Feb 18;7:48. doi: 10.1186/s40425-019-0531-2 (PMC6380053; doi:10.1186/s40425-019-0531-2)
Supplement: Supplementary file 2 — HMG2D Ab is specific for NKG2D. (PDF 110 kb) [file 40425_2019_531_MOESM2_ESM.pdf]

Additional File 2

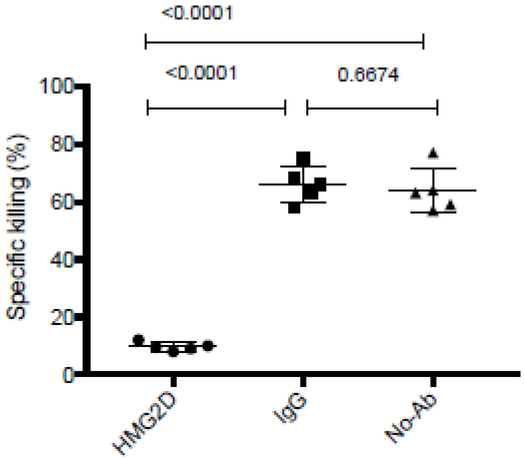

Additional File 2: The effects of anti-NKG2D antibody clone HMG2D are specific for NKG2D. As in Figure 1, at day 0, mice were immunized with peptide-loaded DC subcutaneously and injected retro-orbitally with purified pMel CD8 T cells. One week after immunization, half of the mice were injected intraperitoneally with the anti-NKG2D blocking antibody (Ab) or hamster IgG control (500 ug/dose; BioXCell) and an in vivo CTL assay was conducted the following day. Memory recall responses were analyzed at one month later by repeating the in vivo CTL assay. Data shown are representative of one experiments.
