# Supplementary material for: NKG2D signaling certifies effector CD8 T cells for memory formation
Source: J Immunother Cancer. 2019 Feb 18;7:48. doi: 10.1186/s40425-019-0531-2 (PMC6380053; doi:10.1186/s40425-019-0531-2)
Supplement: Supplementary file 3 — The anti-NKG2D antibody clone HMG2D did not deplete NKG2D expressing cells. (PDF 120 kb) [file 40425_2019_531_MOESM3_ESM.pdf]

Additional File 3

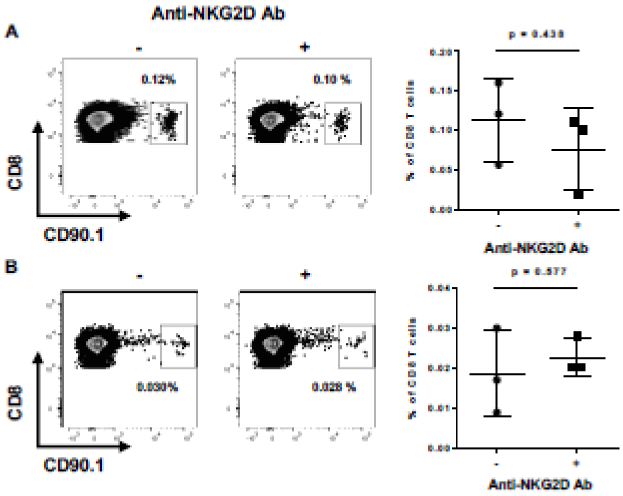

Additional File 3: The anti-NKG2D antibody clone HMG2D did not deplete NKG2D expressing cells. Effector pMel CD8 T cells were generated as described in Fig. 1A. On day 6, half of the mice were injected with the anti-NKG2D antibody clone HMG2D. The percentage of pMel CD8 T cells among the total CD8 splenocytes was analyzed one (**A**) or eight (**B**) days later by flow cytometry. Data shown are representative of two independent experiments.
