# Supplementary material for: NKG2D signaling certifies effector CD8 T cells for memory formation
Source: J Immunother Cancer. 2019 Feb 18;7:48. doi: 10.1186/s40425-019-0531-2 (PMC6380053; doi:10.1186/s40425-019-0531-2)
Supplement: Supplementary file 4 — The absence of NKG2D during the effector phase did not alter the phenotype of the memory CD8 T cells. (PDF 120 kb) [file 40425_2019_531_MOESM4_ESM.pdf]

Additional File 4

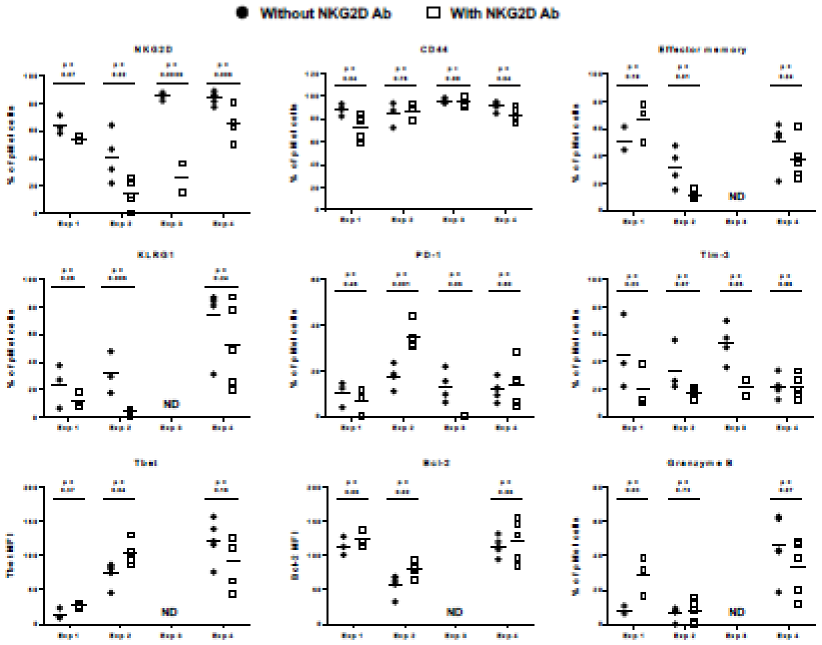

Additional File 4: The absence of NKG2D during the effector phase did not alter the phenotype of the memory CD8 T cells. Memory pMel CD8 T cells were generated as in Fig. 1A, in presence (closed symbols) or absence (open symbols) of NKG2D signaling. Graphs summarize the phenotype of memory pMel CD8 T cells obtained in four independent experiments (Exp). Phenotype was analyzed on CD90.1 (pMel) gated cells present in the spleen of immunized mice one day after in vivo CTL assay. Shown are the percentages of pMel CD8 T cells expressing NKG2D, CD44, PD-1, Tim-3, granzyme B, or KLRG1 and the mean fluorescence intensity (MFI) of Tbet and Bcl-2. Effector memory cells were defined as [illegible]
