# Supplementary material for: NKG2D signaling certifies effector CD8 T cells for memory formation
Source: J Immunother Cancer. 2019 Feb 18;7:48. doi: 10.1186/s40425-019-0531-2 (PMC6380053; doi:10.1186/s40425-019-0531-2)
Supplement: Supplementary file 6 — Endogenous effector CD8 T cells have a slightly decreased effector response in the absence of NKG2D signaling. (PDF 157 kb) [file 40425_2019_531_MOESM6_ESM.pdf]

Additional File 6

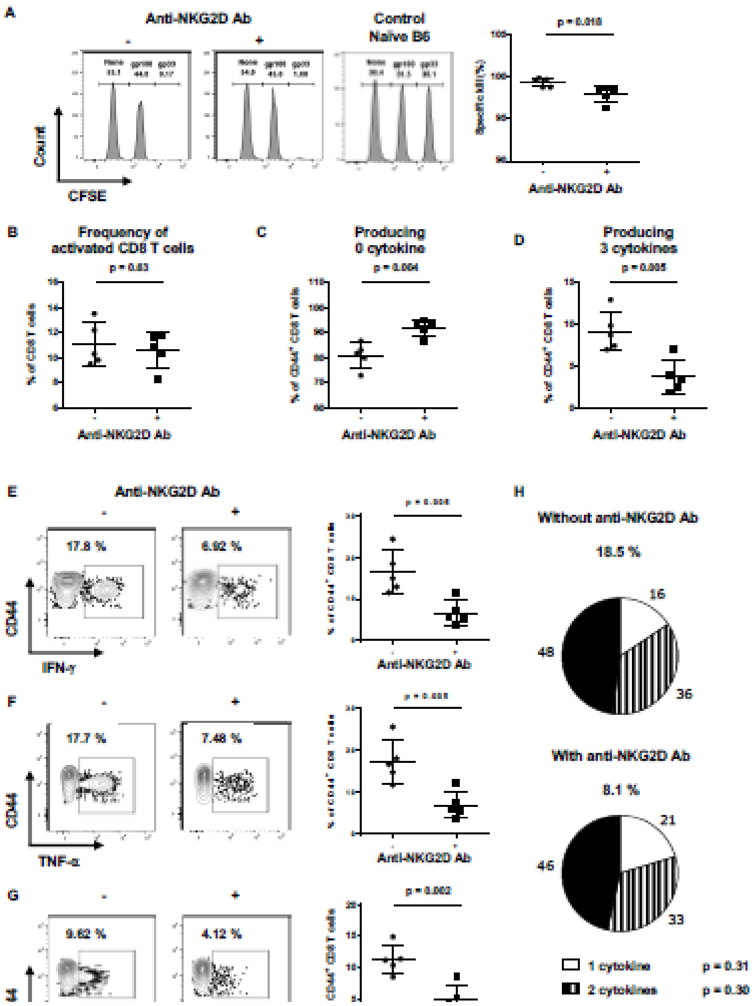

Additional File 6: Endogenous effector CD8 T cells have a slightly decreased effector response in the absence of NKG2D signaling. (A) Example of in vivo CTL assay readout by flow cytometry during effector responses. (B) Representative graph shows the percentages of endogenous antigen-experienced (CD44$^+$) CD8 T cells among total CD8 T cells present in the spleen of immunized mice one day after target injection for in vivo CTL assay. (C-H) Splenocytes from (b) were restimulated overnight with gp33 peptide or irrelevant peptide (hgp100). Cytokine production was measured the next day by flow cytometry. The percentages of endogenous CD44$^+$ CD8 T cells that produce 0 (c) or 3 cytokines (D) are shown. (E-G) Shown are flow examples and graph summarizing the percentage of endogenous CD44$^+$ CD8 T cells secreting IFN-g (E), TNF-a (F) or IL-2 (G). (H) Pie charts show the percentage of endogenous CD44$^+$ CD8 T cells that produce 1, 2, or 3 cytokines among the cells that produce at least one cytokine (denoted above each pie chart). Data shown are representative of two independent experiments.
