# Supplementary material for: NKG2D signaling certifies effector CD8 T cells for memory formation
Source: J Immunother Cancer. 2019 Feb 18;7:48. doi: 10.1186/s40425-019-0531-2 (PMC6380053; doi:10.1186/s40425-019-0531-2)
Supplement: Supplementary file 8 — Memory cells formed upon transient NKG2D blockade were not protective against melanoma B16 tumor. (PDF 118 kb) [file 40425_2019_531_MOESM8_ESM.pdf]

Additional File 8

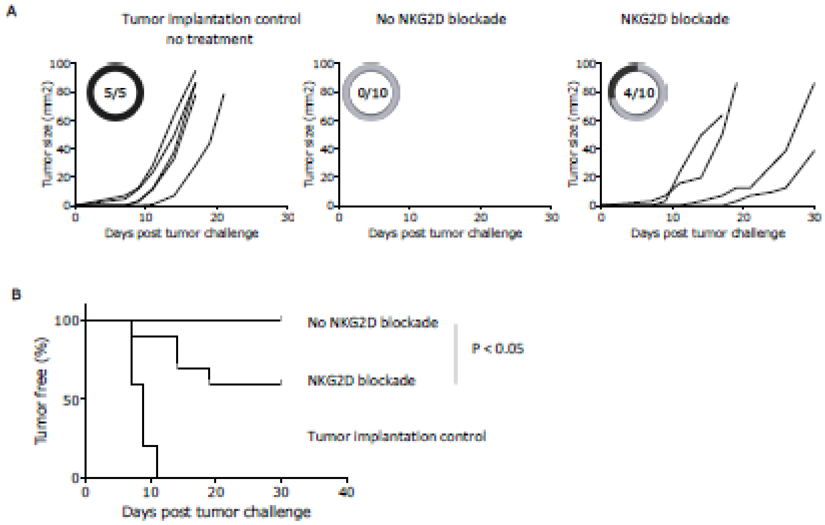

Additional File 8: Memory cells formed upon transient NKG2D blockade were not protective against melanoma B16 tumor. Memory pMel CD8 T cells were generated as in Fig 1A. (**A-B**) B16 melanoma cells were injected during memory phase (>d40). Tumor growth (**A**) and incidence (**B**) were followed over time. Lines in graphs shown in A represent the tumor growth of a single mouse. Tumor incidence in mice with memory pMel CD8 T cells formed in absence or presence of transient NKG2D blockade is indicated on each graph. Naïve C57BL/6 mice were used as control. The graphs show the results of one of two experiments.
