# Supplementary material for: NKG2D signaling certifies effector CD8 T cells for memory formation
Source: J Immunother Cancer. 2019 Feb 18;7:48. doi: 10.1186/s40425-019-0531-2 (PMC6380053; doi:10.1186/s40425-019-0531-2)
Supplement: Supplementary file 7 [file 40425_2019_531_MOESM5_ESM.docx]

Additional File 5

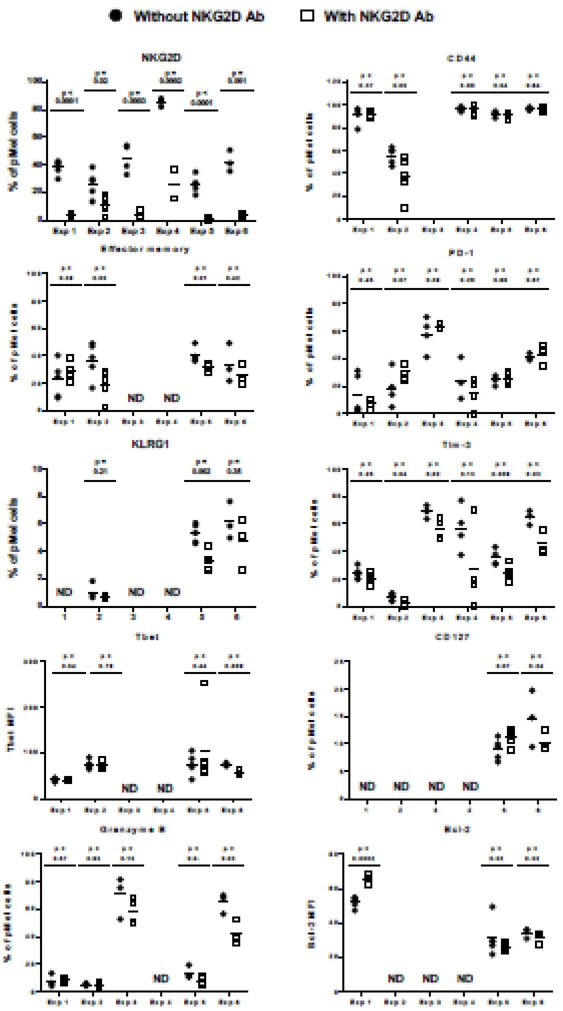

Additional File 5: Blocking NKG2D did not alter the phenotype of effector CD8 T cells. Effector CD8 pMel T cells were generated as described in Fig. 1a. Phenotype of effector CD8 pMel T cells was analyzed one day after in vivo CTL assay, in presence (closed symbols) or absence (open symbols) of NKG2D signaling. Graphs summarize the phenotype of effector pMel CD8 T cells obtained in four independent experiments (Exp). Phenotype was analyzed on CD90.1 (pMel) gated cells present in the spleen of immunized mice one day after in vivo CTL assay. Shown are the percentages of pMel CD8 T cells expressing NKG2D, CD44, PD-1, Tim-3, granzyme B, KLRG1 or CD127 and the mean fluorescence intensity (MFI) of Tbet and Bcl-2. Effector memory cells were defined as $CD44^{+}CD62L^{-}$ cell. ND means not done.
