# Supplementary material for: NKG2D signaling certifies effector CD8 T cells for memory formation
Source: J Immunother Cancer. 2019 Feb 18;7:48. doi: 10.1186/s40425-019-0531-2 (PMC6380053; doi:10.1186/s40425-019-0531-2)
Supplement: Supplementary file 8 [file 40425_2019_531_MOESM7_ESM.docx]

Additional File 7

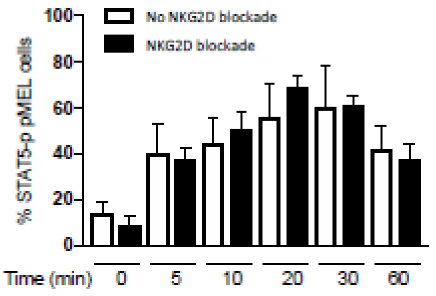

Additional File 7: IL-15-mediated STAT5 phosphorylation remains intact in CD8 T cells that had NKG2D blocked during the effector phase. Effector pMel CD8 T cells were generated as described in Fig. 1A. Shown are the representative plots of pSTAT5 graphs summarizing the percentage of pMel CD8 T cells (CD90.1+) expressing pSTAT5 after ex vivo exposure to IL-15 at different time points (min). This experiment was conducted using 4-5 mice per group. No statistical differences were observed (p>0/05) when comparing both groups at different time points.
